# Shoot Extracts from Two Low Nodulation Mutants Significantly Reduce Nodule Number in Pea

**DOI:** 10.3390/plants9111505

**Published:** 2020-11-06

**Authors:** Christian A. Huynh, Frédérique C. Guinel

**Affiliations:** Department of Biology, Wilfrid Laurier University, Waterloo, ON N2K 3V3, Canada; huyn9220@mylaurier.ca

**Keywords:** autoregulation of nodulation (AON), E107 (*Psbrz*) mutant, E132 (*Pssym21*) mutant, nodulation efficiency, nodule distribution, *Pisum sativum*, plant return on nodule construction cost, shoot-derived inhibitor

## Abstract

E107 and E132 are pea mutants that nodulate poorly. Because they have a shoot-controlled nodulation phenotype, we asked if their mutated genes were implicated in the autoregulation of nodulation (AON), a mechanism which consists of two systemic circuits, the positive CEP/CRA2 and the negative CLE/SUNN, coordinated via NIN and miR2111. We further characterized the mutants’ phenotype by studying nodule distribution and nodulation efficiency. E107 was similar to wild-type (WT) in its nodule distribution, but E132 had an extended nodulation zone with nodules forming distally on its lateral roots. Moreover, we tested whether their shoots produced a compound inhibitory to nodulation. We made ethyl-acetate extracts of roots and shoots of both mutants and WT, which we applied to rhizobia-inoculated WT seedlings and to pure rhizobial cultures. Whereas free-living bacteria were unaffected by any of the extracts, WT treated with shoot extracts from either inoculated mutant had fewer nodules than that of control. E107 and E132 shoot extracts led to a 50% and a 35% reduction in nodule number, respectively. We propose that E107 and E132 belong to a new sub-class of AON mutants, i.e., hypo-nodulators, and that their respective gene products are acting in the AON descending branch, upstream of TML signaling.

## 1. Introduction

Our knowledge on autoregulation of nodulation (AON) has expanded greatly in the last few years [1,2,3]. This regulatory mechanism, highly conserved among legumes, e.g., [4,5], involves a complex and finely-tuned long-distance signaling. Earlier, it was considered solely as a negative feedback pathway, but now it is thought to be comprised of two complementary systemic pathways, e.g., [6,7]. In *Medicago truncatula*, the negative pathway acting through the CLE/SUNN module is counterweighed by the positive pathway acting through the CEP/CRA2 module [7,8]. Together, these two circuits regulate nodule development on a root system so that N_2_ fixation and C demand are optimally balanced within the legume plant In short, upon recognition by the root of the rhizobial -Nod factors, the transcription factor NIN binds to the gene promoters of members of both the CEP and CLE families, activating the production of these two types of short peptides [4,8]. The CLE peptide is modified later, post-translationally, as it becomes arabinosylated by the enzyme encoded by *MtRDN/LjPLENTY/PsNOD3* [5,9]. Upon their synthesis and potential modification in the legume roots, CEP and CLE peptides are transported via the xylem to the shoot, where they are perceived by MtCRA2 and MtSUNN/LjHAR1/PsSYM29, respectively [10,11]. Both types of receptors are leucine-rich repeat receptor-like kinases [6,11]. Once activated, the kinases are thought to act on miR2111 [7], a mobile miRNA molecule known to target for degradation the transcripts of TML [12] that code for Kelch-repeat F-box proteins [13]. TML, localized in the root, likely acts in the proteasome-mediated degradation of its yet-unknown target protein [13]. In effect, the levels of miR2111 appear to be modulated by the action of the two kinases. On one hand, triggering CRA2 results in an accumulation of miR2111 and therefore in lower levels of TML transcripts, leading to the lifting of nodulation inhibition [7]. On the other hand, triggering SUNN prevents miR2111 accumulation and thus increases TML transcript levels, leading to the inhibition of further nodule formation [12,13].

Legume mutants have been essential tools to untangle the AON regulatory mechanism. Involved proteins have been identified via classic forward genetics, and their location of action determined by reciprocal grafting. For example, the study of the *Ljhar1* and *Pssym29* mutants led to the discovery of the CLV1-like receptor kinase in the shoot [11], when that of the *Ljtml* mutant led to the finding of TML, the only player of the AON descending branch known to date to be localized in the root [14]. Up until recently, most mutants studied in the context of AON have been mutants that form abnormally high numbers of nodules [15]. This changed with the characterization of the *cra2* mutants [6] known to exhibit a reduced number of nodules when compared to wild-type (WT); the study of this mutant led to the discovery of the CEP/CRA2 positive systemic pathway. Here, we are focusing on two pea mutants, E107 (*Psbrz*) [16] and E132 (*Pssym 21*) [17], which also have a low nodulation phenotype. Similar to the *cra2* mutant [6], these two monogenic recessive mutants, obtained via ethyl-methane-sulfonate mutagenesis [18,19], have their nodulation phenotype shoot-controlled [17,20]. As well, and similar to the *cra2* mutant [6], E107 and E132 are pleiotropic mutants that exhibit atypical root architecture. Whereas E107 forms fewer and shorter lateral roots (LRs) than WT [21], E132 displays a bushy root system with many more tertiary roots than WT [17]. As for the *cra2* mutant, it exhibits a compact root system with numerous short LRs branching from a short primary root [6]. Despite sharing common traits, relying solely on the root architecture, it is obvious that the three mutants are distinct.

Our objectives were multifold in this study. Knowing that mutants defective in AON display an aberrant root architecture and exhibit an enlarged nodulation zone [22], we set out to characterize the symbiotic root development of, and the nodule distribution in, the mutants E107 and E132. Furthermore, because of their low shoot-controlled nodulation phenotype, we set out to test whether or not these two mutants produce a shoot-derived inhibitory substance. We found E107 and E132 to be distinct both in their symbiotic root architecture and their nodule distribution; moreover, their shoot extracts were effective in reducing the number of nodules formed on WT, with E107 shoot extracts producing a stronger inhibitory effect than E132.

## 2. Results

### 2.1. Detailed Characterization of Nodulation Phenotype 

#### 2.1.1. Root Architecture of Nodulated Plants

**Figure 1:**
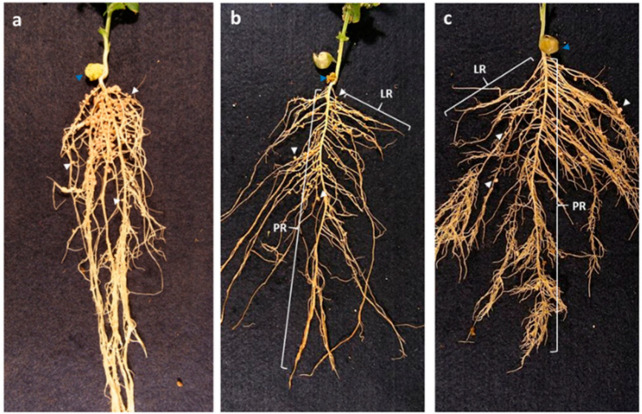
The root phenotypes of the different pea lines inoculated with *Rhizobium leguminosarum*. The images are of roots of wild-type (WT) (**a**), E107 (**b**), and E132 (**c**) harvested 42 days after inoculation. Lateral roots (LR) from the mutants have been pulled away from the primary root (PR) so that nodules (white arrowheads) could be easily distinguished. The blue arrowheads point at the shriveled cotyledons.

E107 and E132 could easily be recognized from WT by their root system (Figure 1). E107 was characterized as having fewer and shorter lateral roots than that of WT (Figure 1b) whereas E132 was recognized by having shorter but more numerous tertiary roots than that of WT (Figure 1c).

Under our growth conditions, the primary root (PR) of each pea line reached its full length at about 21 days after inoculation (DAI). Whereas E107 exhibited a PR length similar to that of WT, E132 had a significantly shorter PR (Table 1). Both mutants had longest secondary roots (LSRs) that were significantly shorter than that of WT, with E132 having the shortest (Table 1). However, while the LSR of E107 increased with age as did that of WT, the LSR of E132 remained short, suggesting that LR growth may be delayed in E107 but halted in E132. This block of growth in E132 secondary roots likely promotes the growth of its tertiary roots. In our growth conditions, these tertiary roots emerged in the most distal portion of the secondary roots (Figure 1c), giving a bushy appearance to the tip of these roots.

#### 2.1.2. Nodule Distribution and Nodulation Efficiency

As expected, the two mutants nodulated poorly. E107 and E132 had, respectively, ten and five times fewer nodules than WT (Table 1). As estimated by their dry weight (DW), nodules on all pea lines increased in size over time; however, whereas WT and E107 nodules reached a peak in their DW at 35 DAI, E132 nodules were the largest at 42 DAI. Both mutants exhibited larger nodules than WT with the E132 nodule DW being significantly higher than that of WT (Table 1). Nodules on both mutants were pink and they hosted bacteroids similar in their Y-shape to those found in WT nodules (data not shown). Furthermore, rhizobia recovered from nodules of both mutants were capable of re-infecting WT roots (data not shown). These observations indicate that nodules not only developed properly in both mutants, but they also provided a functional niche where rhizobia effectively fixed nitrogen and reproduced.

In WT, the nodules were restricted to the area closest to the cotyledons in the crown region of the root system (Figure 1a). Along the PR, nodule coverage started roughly 0.5 cm below the cotyledons and extended downwards about 4.5 cm; moreover, at most times examined, nodules were found in the proximal portion of the LRs (Figure 1a). This area of highest nodule density remained roughly in the same position throughout WT development. Notwithstanding differences in nodule number and root length, the distribution of nodules on E107 was similar to that of WT, i.e., the area of highest nodule density was observed in the crown region of the root system near the cotyledons (Figure 1b). This area of high nodule density was distinctly recognizable as early as 21 DAI, and it remained of the same size and at the same position at all times thereafter. E132 nodule distribution was different from that of WT and E107 (Figure 1c). It was much more extended than in the other two pea lines; in that mutant, at 21 DAI, nodules could be found further down in the distal portions of the PR and LRs. Moreover, the area covered by the nodules in E132 tended to increase with time. 

We chose to estimate the nodulation efficiency of the different pea lines by calculating their plant return on nodule construction cost [23]. A lower value indicates a larger return and a higher efficiency of symbiosis, i.e., the lower the cost of developing a nodule is for the plant, the more efficient is the symbiosis. Not surprisingly, the most efficient pea line in terms of nodulation was the WT; at all ages, its plant return had the lowest value when compared with that of the two mutant lines (Table 1). However, as the WT plants got older, the plant return on nodule construction cost increased significantly (Table 1), suggesting that nodulation efficiency decreased; this decrease over time may reflect nodule senescence in ageing plants. As expected, the two mutants had a higher return, i.e., lower nodulation efficiency, than WT (Table 1). At 21 DAI, the age at which the WT was the most efficient, plant return on nodule construction cost for E107 and E132 was about four and eight times higher, respectively, than that for WT. The two mutants differed nonetheless in that plant return for E107 tended to increase with age, whereas that for E132 tended to decrease (Table 1), suggesting that over time while E107 symbiosis was getting less efficient (as was that of the WT), that of E132 was becoming more efficient. This difference is likely related to nodule distribution as on the older E132 plants, several nodules were located distally on the root system (Figure 1c); these younger nodules that formed later would still be efficient at fixing nitrogen.

**Figure 2:**
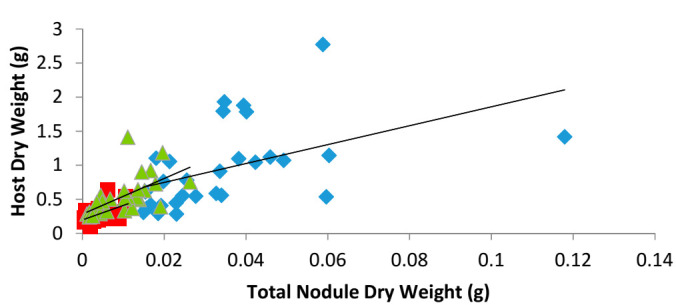
Relationships of host dry weight and nodule dry weight in the different pea lines; WT plants are represented by blue diamonds, E107 plants by red squares, and E132 plants by green triangles. Plants were harvested throughout their development (at 14, 21, 38, 35, and 42 DAI), and data from all ages were taken into consideration (i.e., *n* = at least 24 for each pea line) to perform the regression analysis.

Another approach to assess the symbiotic efficiency was to perform a regression analysis whereby the relationship between total nodule DW and host DW, as independent and dependent variables, respectively, was studied (Figure 2). We reasoned that this analysis would give us a better understanding of the mutants’ symbiotic relationship. In brief, the correlation coefficient Spearman’s ρ was calculated for each pea line and transformed by Fisher z-transformation; a Z-test was then used to determine whether the correlations were significantly different from each other. We found that the two parameters were indeed correlated for each pea line (WT, ρ = 0.656; E107 ρ = 0.45; E132 ρ = 0.782), and that this correlation was significant (*p* < 0.05). A line of best fit was drawn to illustrate better these correlations. Furthermore, when comparing the mutants to the WT, we found that host DWs and nodule DWs were similarly correlated (E107 to WT, z-value = 1.052; E132 to WT, z-value = 0.945; Figure 2).

### 2.2. Effect of Crude Plant Extracts on WT Symbiosis

**Figure 3:**
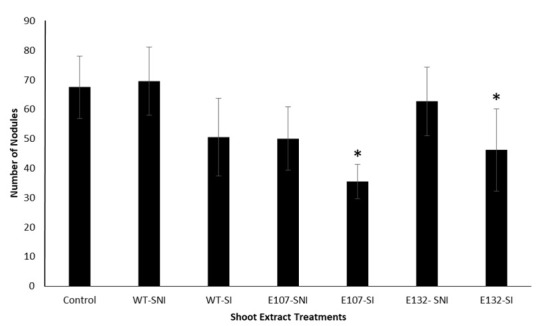
The effect of shoot extracts on WT nodulation. Inoculated WT plants were treated with the extracts derived from shoots of WT, E107, or E132 plants, inoculated (SI) or not (SNI) with rhizobia. Water was used as the control treatment. Nodules were counted at 21 DAI. Values represent means ± standard errors, *n* = 15. An asterisk denotes significant differences to the control.

To test whether a compound inhibitory to nodulation was synthesized by the mutants, ethyl acetate extracts were made from the shoots and roots of the three pea lines, and from both inoculated and non-inoculated plants. All extracts were then tested on WT nodulation. Extracts obtained from the roots of all pea lines, whether the seedlings had been inoculated or not, had no effect on WT nodulation (data not shown), i.e., the number of nodules formed on treated WT plants was not significantly different from that found on WT control plants. In contrast, the extracts derived from the shoots of the inoculated mutant pea lines were inhibitory to nodule formation as there were significantly fewer nodules on WT treated with these extracts than on control WT or on WT treated with shoot extracts obtained from non-inoculated plants (Figure 3). The shoot extract of inoculated E107 plants had the strongest effect because the nodule number seen on WT plants was reduced by half when compared to that seen on WT having received the water treatment. The extract of inoculated E132 shoots was less potent as it reduced nodule number on WT by only a third (Figure 3).

To determine whether this inhibitory effect was a plant or a bacterial response to the shoot extract, a bacterial viability assay was performed. No significant differences in abundance were found between the culture treated with water (control; 9.55 × 10^8^ ± 2.08 × 10^8^ colony-forming units (CFU)/mL) and those treated with the shoot extracts derived from non-inoculated or inoculated plants (data not shown). These results suggest that the inhibitory effect of the shoot extracts derived from the inoculated mutants was a plant response and not a bacterial response. 

Because at 21 DAI individual nodule DW was higher in E132 than that in WT (Table 1), we measured the DW of the nodules of plants treated with the different shoot extracts to determine whether this parameter was affected by the treatment. Only nodules of WT plants treated with the shoot extracts obtained from inoculated E107 plants were significantly affected. In that particular treatment, individual nodule DW increased five-fold from 0.046 ± 0.006 mg (control treatment) to 0.247 ± 0.092 mg (plants treated with E107 extracts), suggesting that the E107-derived shoot extract had a stimulatory effect on nodule meristem growth.

## 3. Discussion

Despite exhibiting similar low nodulation and shoot-controlled phenotypes [16,17,20], the mutants E107 (*Psbrz*) and E132 (*Pssym21*) are clearly distinct. Thus, both are affected at different timing in their nodule development. In E107, nodulation is affected early at the interface of the epidermis–cortex [16], whereas in E132, nodulation is affected later during nodule emergence [17]. Furthermore, whereas E132 has a mycorrhizal phenotype similar to that of WT [24], E107 forms a low number of colonization units, with most fungal entry prohibited beyond the epidermis [20]. Here, we have further characterized the nodulation phenotype of these mutants. We have shown that neither of them is compromised in its symbiotic efficiency. Both are capable of offering a functional niche to the micro-symbionts, and their symbiosis efficiency as assessed by regression analysis do not appear to be affected by their respective mutation. Indeed, in the mutants as in WT, host DW and total nodule DW were tightly correlated and the correlations were similar. A different picture was obtained when considering plant return on nodule construction costs to estimate nodulation efficiency; in that instance, both mutants had significantly higher costs of constructing a nodule than that of WT. This may suggest that this parameter, used previously to compare bacterial mutants and assess their symbiotic efficiency [23], is not a useful parameter to estimate symbiosis efficiency in legume mutants. 

Our most interesting result is that both mutants possess a shoot signal which is inhibitory to the process of nodule development. This is, to our knowledge, the first time that low nodulation mutants are shown to possess an inhibitor made in the shoot and acting on the root. Moreover, our data suggest that this compound is (1) a response to the presence of rhizobia since shoot extracts from non-inoculated plants are not effective on WT nodulation, and (2) polar since it is soluble in water. Several studies have been performed in search of such a shoot-derived signal and it is possible that more than one exists [25]. Markwei and LaRue [17] reported that in pea it could be an anion or a neutral molecule with a molecular mass lower than 1000. Lin et al. [26] characterized a signal in soybean as being heat-stable, less than 1 kDa in size, and resistant to RNase-A and Proteinase-K enzymes. Phytohormones as well have been proposed as signals, and both jasmonic acid [27] and cytokinin [28] have been suggested as candidates. Since the signals are unknown, their downstream targets in the AON descending branch are yet to be deciphered; however, pieces of the puzzle are constantly being uncovered, e.g., [29].

The existence of this inhibitory substance makes these two mutants clearly distinct from *cra2* mutants, the low nodulation phenotype of which is caused by the disruption of the positive CEP/CRA2 control of nodulation [6,7,8]. This raises a few questions, and an obvious one is to which of the two circuits making the AON pathway are E107 and E132 participating. Reciprocal grafting experiments performed by Markwei and LaRue [17] may help answer this question for E132. Not only did these authors graft E132 with WT, grafts that led to the discovery of the E132 shoot-controlled nodulation phenotype, but they also grafted E132 with the *Psnod3* super-nodulator mutant. If NOD3 and SYM21 belonged to a single pathway, then one would expect both E132/*nod3* (scion/stock) grafts and *nod3*/E132 grafts to exhibit a nodulation phenotype similar to that of WT. Interestingly, the prediction is correct for the E132/*nod3* graft, suggesting that NOD3 and SYM21 are part of one single circuit activated by the recognition of the CLE peptides by SUNN. However, the prediction is incorrect for the *nod3*/E132 grafts, where the E132 stock bore a number of nodules significantly higher than that of WT but significantly lower than that of *nod3* mutants. Markwei and LaRue [17] were unable to explain this result nor the result obtained by grafting WT onto E132. Remarkably, in their experiment, having E132 as a stock appeared to lead to a situation where TML regulation (i.e., repression of nodulation) was lifted. To us, this is evidence that E132 is located upstream of TML, but to place the mutant firmly on the CLE/SUNN circuit will require further work. Markwei and LaRue’s results [17] may indicate the existence of a redundant pathway, whereby, despite the inhibitory effect of the E132 shoot-derived compound, *nod3* would still exert its stimulation on nodulation. No such grafts were made in the case of E107, but it would be interesting to undertake such experiments, especially once CRA2 homologs are discovered in pea. Grafting E107 with *nod3* mutants and with *cra2* mutants should shed light on the circuit in which this mutant is involved. The recent study highlighting the function of the CLE/SUNN module in arbuscular mycorrhizal (AM) colonization [30] may be helpful in understanding E107. This pea mutant forms successful associations with *Rhizophagus aggregatum*, but at a low frequency [20]. Furthermore, E107 root-organ cultures challenged with *R. irregularis* are known to exhibit a hyper-mycorrhizal phenotype (unpublished data). It would be worthwhile to test the effect of E107 shoot extracts on WT peas inoculated with AM fungi. These experiments would place E107 more firmly on the descending branch of the CLE/SUNN circuit and would confirm whether or not that circuit controls both symbioses in pea. In any case, at this point, it is impossible for us to take a strong stand for the location of the two pea mutants in the AON pathway, as there are other possibilities as to their placement. For example, the phenotype of the two mutants may be a reflection of a loss of miR2111 sensitivity, whereby nodulation would not be controlled because the module miR2111-TML would not be turned on. In response, the mutants could over-produce a compound to compensate for the loss.

Taking into consideration the *cra2* mutants and the two pea mutants studied here, we trust that the description and classification of AON mutants should be re-evaluated and extended. Gresshoff et al. [22] defined AON mutants based on their high nodule number, extended nodulation zone, increased nodule DW, insensitivity to nitrate, and less developed root system. Novak [15] refined this classification by proposing two different categories of mutants. In his view, hyper-nodulating mutants are nitrate-sensitive and ethylene-insensitive, and their nodules form in a zone similar to that of WT; in contrast, super-nodulating mutants are nitrate-insensitive and ethylene-sensitive, and their nodules emerge outside the initial zone of nodulation [15]. According to these definitions, the mutants studied here are not AON mutants. Nonetheless, all traits used to define these mutants are affected one way or another in E107 and E132, as well as in *cra2* mutants. The two pea mutants form a low number of nodules, which are of a larger size than those of WT. Moreover, whereas E132 has a more extensive nodulation zone than WT and E107, it exhibits a much more developed root system. Both mutants are sensitive to ethylene [16,17]; however, to our knowledge, their sensitivity to nitrate was never tested, and yet it would be informative. As for the *cra2* mutants, they form a low number of nodules, which are of a similar weight to WT nodules, are sensitive to nitrate, and have a reduced root system [10]. They have been characterized as ethylene-sensitive [31]. In effect, the protein EIN2 gets phosphorylated by the CRA2 kinase [32], but when phosphorylated, EIN2 is inactive, resulting in the inhibition of the ethylene signaling pathway and consequently in the promotion of nodulation [33]. In light of the findings on these low nodulating mutants, we propose to add another category to the AON mutants, i.e., that of hypo-nodulators; the *cra2*, E107, and E132 mutants would belong to that group. This sub-class is not homogenous, and it likely comprises other mutants.

Recently, in the literature, different groups have highlighted the need to identify the shoot-derived signal(s) inhibitory to nodulation [25,29]. We think that E107 and E132 would be excellent tools to use as a starter in the quest of these signals. Identifying the products of the two genes would establish whether or not there are one or two compounds involved. The recent publication of the pea reference genome [34] may facilitate the identification of these compounds. We predict that the proteins encoded by *PsBRZ* and *PsSYM21* are acting as two distinct downstream effectors in the AON regulatory pathway. At this point, it is difficult to place the two mutants in the pathway; however, we feel confident in placing them upstream of *TML*. 

## 4. Materials and Methods 

### 4.1. Plant Growth Conditions 

Seeds of *Pisum sativum* cv. Sparkle (wild type, WT) and of its two mutants E107 (*Psbrz*) and E132 (*Pssym21*) were surface-sterilized [35]. Depending on the experiments, imbibed seeds were planted in Conetainers™ (55 mL or 656 mL; Stuewe & Sons, Tangent, Oregon, USA) or in square plastic pots (560 mL) but always in sterile vermiculite (Holiday, Vil Vermiculite Inc., Toronto, ON, Canada). Planted seeds were placed in a temperature- and photoperiod-controlled growth room (23 °C/18 °C; 16 h/8 h; light intensity of 120 to 150 μE m^−2^s^−1^). The seedlings were sub-irrigated with water for the first 10 days; afterwards, at every third watering, non-inoculated plants received a complete nutrient solution whereas inoculated plants were given a low-nitrogen nutrient solution [35].

When needed, *Rhizobium leguminosarum* bv. *viciae* 128C53K was grown from cultures maintained on yeast-mannitol agar stored at −20 °C. Bacterial cultures were incubated at 25 °C in an orbital shaking water-bath for 48 h at 100 rpm. Cultures were grown until they reached the stationary growth phase as determined by an absorbance reading between 0.8 and 1.1 at 600 nm on a spectrophotometer. Three days after planting, each seedling was flood-inoculated with 5 mL of a 5% bacterial aqueous solution. 

### 4.2. Nodule Distribution and Nodulation Efficiency 

A developmental study was performed where six plants grown in large Conetainers™ were randomly harvested at 14, 21, 28, 35, and 42 days after inoculation (DAI). At each harvest time, shoots were separated from roots and root architecture was carefully observed; the lengths of the primary root (PR) and of the longest secondary root (LSR) were measured. The position of nodules was recorded in relation to these two roots [24,36], and their number counted. Shoots, roots, and nodules were placed individually in a drying oven set at 60 °C to measure, three days later, their dry weights (DW). The experiment was repeated three times for a total of 18 plants.

Plant return on nodule construction cost (i.e., host plant DW divided by total nodule DW) [23] was calculated to evaluate symbiotic efficiency. To obtain a global view of the cost of developing a nodule, data obtained from individual plants across all harvest times were pooled and a regression for each pea line was created. Nodule DW (the independent variable) was used as a measurement of the plant investment and host DW (the dependent variable) as an estimation of the return on the plant investment [23].

### 4.3. Assessing the Effect of Crude Plant Extracts on WT Symbiosis 

#### 4.3.1. Making the Crude Extracts 

Seeds of each pea line were planted, one per square pot, and half of the seedlings were inoculated. All plants were harvested 24 days after planting, i.e., inoculated plants were harvested 21 DAI. On harvest day, it was ensured that no contamination had occurred between non-inoculated and inoculated plants. Shoots and roots were separated, weighed, chopped, and individually placed in 50 mL conical centrifuge tubes. Samples from the same pea line were pooled and covered with ~20 mL of ethyl acetate; they were left to macerate at 4 °C in the dark for 72 h at which time the ethyl acetate was removed and placed in 250 mL round-bottom flasks. A volume of sterile water equal to the weight of the tissue (v:w) was added to the maceration fluid, which was then evaporated under a stream of argon. The aqueous extract left behind was then filter-sterilized using a PES syringe filter with a pore size of 0.2 μm (VWR International, Radnor, PA, USA). The different extracts were either used immediately or stored in 50 mL conical tubes at −20 °C for later use. Before use, the extracts were diluted 20 times in sterile water and on the treatment days, 3 mL of the diluted solution were applied at the soil surface. 

#### 4.3.2. Nodulation Bioassay with Plant Crude Extracts

WT seeds were planted individually in small Conetainers™ and seedlings were inoculated with rhizobia as described above. One DAI, the seedlings were treated with sterile water as a control or with the different ethyl acetate extracts (i.e., WT, E107 or E132 extracts from either shoots or roots); furthermore, extracts of both inoculated and non-inoculated plants were tested. WT plants were similarly treated every 3 days thereafter until harvest time, i.e., 21 DAI. Nodules were counted, excised, and pooled before their DW was determined. This value was used to obtain an individual nodule DW. In a single experiment, five WT seeds were tested per treatment, and the experiment was repeated three times for a total of 15 plants. 

#### 4.3.3. Bacterial Viability Assay with Plant Shoot Extracts

To assess whether the shoot extracts had an effect on rhizobial growth, *R. leguminosarum* was grown in liquid culture supplemented with the different extracts, and bacterial viability was determined. For each treatment, 100 μL of bacterial culture, 150 μL of the extract (20 times dilution), and 2.75 mL of yeast-mannitol broth were placed in round-bottom vented cell culture tubes (BD Biosciences, Franklin Lakes, NJ, USA). An additional treatment with distilled water was used as a control. Bacterial cultures were grown at 25 °C until they reached the stationary growth phase. The abundance of colony-forming units (CFU) was determined on yeast-mannitol agar plates via a dilution series (10^−4^ to 10^−8^). Optimal counts were obtained 48 h after plating (100 μL at 25 °C) using the 10^−8^ dilution. Two independent trials were performed, and for each trial, there were two replicates. For convenience, the results are given as CFU/mL; to do so, the number of CFUs was averaged and multiplied by the dilution factor. 

### 4.4. Statistical Analyses

For the developmental study, means (± standard errors) were compared between mutants and WT using the Student’s *t*-test (*p* < 0.05). A Mann–Whitney Rank Sum Test was performed where the data failed the normality test. A Kruskall–Wallis test (*p* < 0.05) and a Dunn’s post-hoc test (*p* < 0.05) were performed, respectively, to determine significance between all ages within a single pea line and significance at a single age but between pea lines.

For the regression analysis, Spearman’s ρ, a non-parametric equivalent to the correlation coefficient R2, was used to determine correlation coefficients because the data did not fit with parametric statistical assumptions. Correlation coefficients approaching +1 or −1 were deemed to approach perfect correlation. The significance of the correlations existing between total nodule DW and host DW was assessed by transforming ρ values into z-values via Fisher transformation and using Fisher’s z-test (*p* < 0.05). Using the same test, correlations were compared between the mutants and WT. In order to determine whether this relationship was significantly different between the pea lines, a non-parametric test equivalent to an ANCOVA as described by Quade [37] was performed (i.e., a rank analysis of covariance). 

For the nodulation bioassay with plant extracts, means (±standard errors) were compared between the different treatments. A one-way ANOVA was performed to determine the significance of results between all treatments and a Tukey post-hoc test was used to determine significance to the control. For the plant extract assays on bacterial growth, an ANOVA (*p* < 0.05) was performed to compare all treatments.

Statistical analyses were performed using SPSS Statistics 20 (IBM, Armonk, NY, USA) and with Graphpad Prism 8 (Graphpad, San Diego, CA, USA).

## Figures and Tables

**Table 1:** Quantitative characterization of the nodulated root systems of the wild-type (WT) and the mutants E107 and E132. Values are presented as means ± standard errors (*n* = 6 per age, pea line, and trial; three trials were performed for a total of *n* = 18).

Parameter ^5^	14 DAI ^1^	21 DAI	28 DAI	35 DAI	42 DAI
PR^1^ Length (cm)					
WT	20.2 ± 1.3	23.4 ± 1.5	24.7 ± 1.4	24.1 ± 1.0	21.9 ± 1.2
E107	22.2 ± 1.9	22.6 ± 1.1	20.7 ± 1.7	21.2 ± 0.4 *	23.6 ± 1.1
E132	17.7 ± 1.5 *	17.7 ± 0.9 *	17.4 ± 0.7 *	18.7 ± 0.8 *	19.7 ± 0.8
Length of LSR ^1^ (cm)					
WT	18.2 ± 1.0 ^a^	23.7 ± 0.9 ^b^	25.0 ± 0.9 ^b^	25.3 ± 0.8 ^b^	22.4 ± 1.4 ^b^
E107	14.4 ± 1.3 ^a^*	20.4 ± 1.1 ^ab^*	21.7 ± 1.3 ^b^*	20.2 ± 0.7 ^b^*	18.2 ± 1.4 ^b^*
E132	12.1 ± 0.7 *	12.6 ± 0.7 *	13.2 ± 0.6 *	15.0 ± 0.6 *	15.6 ± 1.0 *
Nodule Number					
WT	81.9 ± 10.4 ^a^	194.0 ± 11.6 ^b^	174.4 ± 18.0 ^b^	190.1 ±13.4 ^b^	220.5 ± 20.6 ^b^
E107	4.5 ± 1.8 ^a^*	19.5 ± 4.5 ^b^*	19.1 ± 2.5 ^b^*	20.7 ± 2.0 ^b^*	24.9 ± 3.6 ^b^*
E132	5.3 ± 2.5 ^a^*	37.3 ± 5.4 ^b^*	37.9 ± 6.4 ^b^*	44.9 ± 3.9 ^b^*	41.8 ± 5.8 ^b^*
Nodule DW ^2^ (mg)					
WT	0.048 ± 0.007 ^a^	0.111 ± 0.010 ^b^	0.151 ± 0.024 ^b^	0.205 ± 0.034 ^b^	0.166 ± 0.024 ^b^
E107	ND ^3^	0.101 ± 0.072	0.170 ± 0.077	0.330 ± 0.130	0.242 ± 0.061
E132	ND ^3^	0.200 ± 0.130 ^a^*	0.132 ± 0.030 ^a^	0.216 ± 0.040 ^ab^	0.499 ± 0.090 ^b^*
Plant Return on Nodule Construction Cost ^4^					
WT	ND ^3^	16.76 ± 1.84 ^a^	19.61 ± 3.06 ^a^	30.16 ± 5.23 ^ab^	47.98 ± 1.97 ^c^
E107	ND ^3^	64.48 ± 14.97	127.95 ± 41.12 *	169.64 ± 76.24 *	119.01 ± 52.64 *
E132	ND ^3^	130.73 ± 28.12 ^a^*	55.41 ± 12.92 ^b^	74.84 ± 13.01 *	60.88 ± 11.98 *

^1^ DAI, days after inoculation; PR, primary root; LSR, longest secondary root. ^2^ This represents the dry weight (DW) of an individual nodule, obtained by dividing the total nodule DW of a plant by the number of nodules on that plant ^3^ Values not determined (ND) since most mutants did not nodulate at that age. ^4^ Plant return on nodule construction cost is calculated by dividing the host plant DW by the DW of all nodules found on the host root system; it is used here as a measure of symbiotic efficiency. ^5^ Significant differences between a specific pea line and WT at a single age are indicated by asterisks. Different letters denote significant differences within a single line of pea across time.
